# Defining intraspecific conservation units in the endemic Cuban Rock Iguanas (*Cyclura nubila nubila*)

**DOI:** 10.1038/s41598-020-78664-w

**Published:** 2020-12-10

**Authors:** Kyle J. Shaney, L. Grisell Diaz-Ramirez, Sayra Espindola, Susette Castañeda-Rico, Vicente Berovides-Álvarez, Ella Vázquez-Domínguez

**Affiliations:** 1 Departamento de Ecología de La Biodiversidad, Instituto de Ecología, Universidad Nacional Autónoma de México, Ciudad Universitaria, 04510 Ciudad de México, México; 2 Division of Geriatrics, Department of Medicine, University of California San Francisco, San Francisco, CA USA; 3 Center for Conservation Genomics, Smithsonian Conservation Biology Institute, National Zoological Park, Washington, DC 20008 USA; 4 Department of Biology, George Mason University, Fairfax, VA 22030 USA; 5 Facultad de Ciencias Biológicas, Universidad de La Habana, Calle 25, # 455, entre J e I, Vedado, Ciudad Habana, Cuba

**Keywords:** Ecology, Conservation biology, Evolution, Evolutionary genetics

## Abstract

Defining conservation units is an important step in species management and requires interpretation of the genetic diversity and ecological function of the taxon being considered. We used the endemic Cuban Rock Iguanas (*Cyclura nubila nubila*) as a model to highlight this challenge and examined patterns of its intraspecific genetic diversity across Cuba. We evaluated nuclear (microsatellite loci) and mitochondrial diversity across eight populations from the island and its off-shore cays, and applied the population genetics results for assignment of Management Unit (MU) status and Evolutionary Significant Units (ESUs) based on phylogeographic and time of divergence information. We identified at least six distinct Cuban Rock Iguana MUs, encompassing demographically isolated and genetically differentiated populations across Cuba, most with low effective population size, declining populations, and with high risk of inbreeding and genetic drift. Hence, each MU should be considered of urgent conservation priority. Given the key ecological seed dispersal role of *C. n. nubila*, the disappearance of any MU could trigger the loss of local ecological functional diversity and major negative impacts on their ecosystems. Two divergent ESUs were also identified, exhibiting an historical east–west geographic separation on Cuba. Based on a Caribbean phylogeographic assessment, our findings strengthen the conclusion that all geographically and evolutionarily differentiated *Cyclura* species and subspecies across the archipelago warrant ESU distinction.

## Introduction

Conservation of biodiversity is considered a top global priority^1^. To combat biodiversity loss, conservation efforts have focused on preserving diversity at several levels, including whole ecosystems, species and genetically distinct intraspecific populations^2^. Nonetheless, conservation of intraspecific genetic diversity has received less attention in policy and application^2,3^. In addition, conservation units (CUs) have been assigned and used in management planning, but variation in their definitions and applications is widespread.

Ryder^4^ first defined Evolutionarily Significant Units (ESUs), with the purpose of identifying independently evolving segments within species^5^. The USA Endangered Species Act uses units similar to ESUs, termed Distinct Population Segments (DPSs,^2,6,7^). Similarly, Green^8^ proposed the concept of Designatable Units (DUs), which are populations that have a different conservation status, genetic distinction, range disjunction, and/or biogeographic distinction. The DU concept has been adopted by the Committee on the Status of Endangered Wildlife in Canada for species assessment under the Canadian Species at Risk Act^8^. Funk et al.^7^ reviewed other units, including Management Units (MUs), explaining that multiple MUs may form a single ESU. Regardless of the type of “unit”, these are all variations of CUs and the overall goal of CUs is to conserve varying levels of intraspecific diversity. Coates et al.^2^ explained that two common goals to conserve population level diversity are: ‘historical’, namely independently evolving segments, or ‘adaptive’, focused on preserving functional ability. Which to prioritize (or both) is, however, situation dependent; the species, ecosystem, region, and politics at hand may dictate how to apply conservation efforts across whatever “units” are defined.

A factor that has recently drawn more attention in wildlife management planning is maintenance of ecological function^9^. Empirical data have shown that not all species serve equal roles in their respective ecosystems (e.g., keystone species,^10^). Brodie et al.^9^ recommended focusing on primary functional roles that (1) prevent extinctions, (2) moderate biogeochemical processes, or (3) support ecosystem processes or stability. Pollination, dispersal, ecosystem engineering, and nutrient cycling are a few examples. Although specific suggestions have been provided on how and where to define conservation units, the ecological function of a species has not often been given enough attention^11^. If a species contributes what managers consider to be a disproportionately important role in its ecosystem, it may merit additional effort to conserve that species. For example, Leclerc et al.^12^ showed that anthropogenic pressures resulted in a loss of unique functions in a high percentage of endemic mammals and birds in insular ecosystems. In a recent study, Zipkin et al.^13^ showed that the decline in frog diversity due to the Chytrid fungus was probably indirectly responsible for the correlated decline in Neotropical snake diversity. The ecological ramifications regarding the disappearance or decline in abundance of a species is case specific, but the consequences can be immense. Thus, a variety of ecological and genetic issues may significantly complicate the recognition of CUs.

The Caribbean island chain is an excellent model for examining complex ecological and evolutionary patterns and processes^14,15^, although those complexities make it a challenging system for conservation. West Indian Rock Iguanas (genus *Cyclura*) of the Caribbean exhibit a high degree of endemism, with only one case of sympatry among recognized taxa^16^. Consequently, they are among the most critically endangered lizards in the world, primarily as a result of habitat degradation, direct human-hunting practices, introduction of invasive species, the population dynamics of small populations, and impending sea level rise^17,18^. Rock Iguanas are the largest native herbivores on these islands, and their high biomass and role in seed dispersion have made them critical for ecosystem function^19^. Many *Cyclura* populations have already disappeared, with a consequent loss of species, genetic diversity, and ecological functions^18,20^. All extant species are considered critically endangered, endangered or vulnerable by the IUCN^21^ and are protected under CITES (Appendix I). Despite their immediate conservation urgency and critical keystone ecological roles, we lack intraspecific genetic data for many Rock Iguana species, while their CU assignment and consideration of their ecological contribution in management strategies is also currently needed^22^.

One species in particular, the endemic Cuban Rock Iguana (*Cyclura nubila nubila*), was widely distributed across Cuba and the sub-archipelagos Sabana-Camaguey, Canarreos, and Jardínes de la Reina. This species has been recognized as having historically high abundance; however, its populations have been declining rapidly^23,24^. It is now classified as vulnerable by the IUCN^25^, although with deep information gaps and thus in urgent need of a new assessment. Moreover, although *C. n. nubila* has been included in a preliminary phylogenetic analysis^20^, and nuclear microsatellite characterization and diversity assessments have been done^26–28^, no comprehensive survey of genetic structure has been performed on wild Cuban populations. Given that the Caribbean archipelago and Cuba are considered to be of high conservation priority (Biodiversity Hotspot and Wildlife Conservation Society (WCS) priority^29^; Critical Ecosystem Partnership Fund (CEPF)^30^), it is imperative that threatened populations within this island system be managed properly, based on solid population and genetic data.

Hence, we evaluated nuclear (microsatellites) and mitochondrial diversity across eight populations of *Cyclura nubila nubila* from Cuba and its off-shore cays. Our objective was to assess genetic diversity and structure within and among *C. n. nubila* populations*,* and use that information as the basis for identifying MUs and ESUs, in order to propose conservation measures for those populations. Considering the island-endemic status of this iguana, its historically widespread distribution across the island, and the current degree of isolation of many of its populations, we predicted that they would have low genetic variability and be highly structured. Based on a broader phylogeographic assessment of all *Cyclura* species, we aimed to strengthen the argument that the Caribbean Rock Iguanas, with their evolutionary and geographic uniqueness, warrant ESU distinction.

## Results

### Genetic population diversity and demography

**Figure 1:**
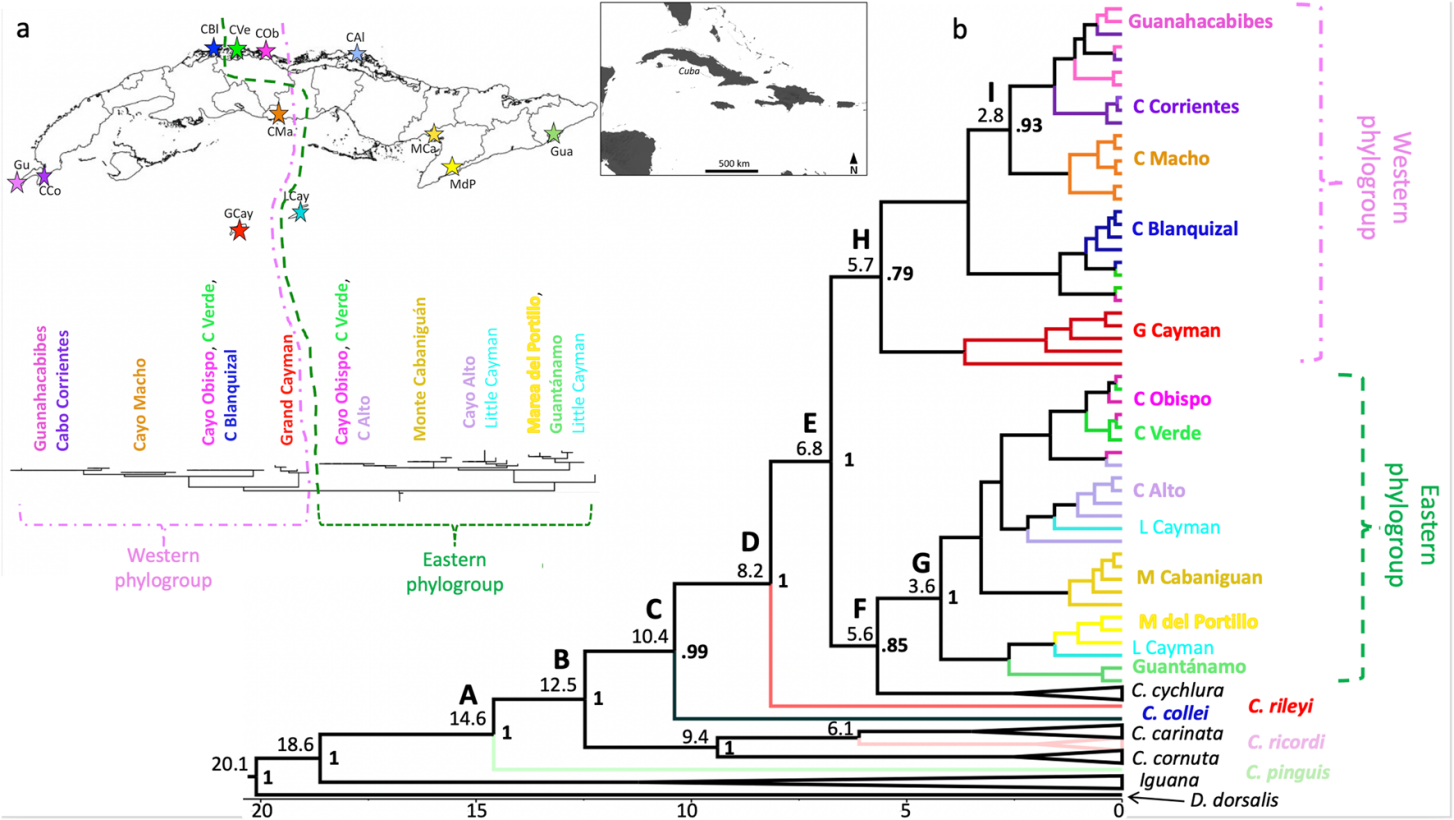
(**a**) Map of sampling localities for *Cyclura nubila nubila* from Cuba (see Supplementary Table S1) on top and a Bayesian species tree (divergence-time) on the bottom, indicating two divergent phylogroups (Western and Eastern). The geographic location of Cuba is shown in the inserted map. Star symbols and matching colors correspond to the eight sampling sites from this study (Gu: Guanahacabibes, CCo: Cabo Corrientes, CBl: Cayo Blanquizal, CVe: Cayo Verde, COb: Cayo Obispo, CMa: Cayo Macho, CAl: Cayo Alto, MCa: Monte Cabaniguán). Two Cuba sites (MdP: Marea de Portillo and Gua: Guantánamo) and the Cayman islands (GCay: Grand Cayman, LCay: Little Cayman) correspond to sequences obtained from GenBank (see Supplementary Fig. S1). (**b**) Divergence-time estimation (time-scale in millions of years; My) of *Cyclura nubila nubila* from Cuba and *Cyclura* species and subspecies across the Caribbean; *Iguana iguana*, *I. delicatissima*, and *Dipsosaurus dorsalis* used as outgroups. Estimated with Beast 1.10.4^78^ and based on the ND4/ND5 mitochondrial region. Numbers above branches are mean divergence time in million years, and at nodes depict posterior probability support. The most basal lineages start on the east side of the archipelago, and follow a west to east distribution: *Cyclura pinguis*, inhabiting Anegada Island, is the most basal haplotype from which two clades are derived, one including *C. cornuta* (Puerto Rico, Dominican Republic), *C. ricordi* (Dominican Republic) and *C. carinata* (Turks and Caicos). The other clade encompasses a basal *C. collei* (Jamaica) haplotype, followed by *C. rileyi* (San Salvador) and a *C. cychlura* (Bahamas) group of haplotypes. Maps were drawn with QGIS v.3.10 (https://qgis.org/en/site/).

We sampled the Cuban Rock Iguana, *Cyclura nubila nubila*, at eight localities across the island of Cuba (Fig. 1a), including a total of 172 individuals (Supplementary Table S1). We amplified 10 microsatellite loci, two of which did not amplify correctly and one that was monomorphic (HDZ35, HDZ66, and HDZ419, respectively). Thus, our genetic analyses were based on seven loci (Supplementary Table S2). No null alleles were consistently identified across loci and sampling localities, most loci were in Hardy–Weinberg equilibrium, and only the pair 152/154 from Cayo Macho showed linkage disequilibrium. We examined genetic diversity for each population (sampling locality) and overall for all populations (Table 1). A total of 141 alleles across loci was obtained, and for each population we calculated the mean number of observed alleles per locus (*N*_a_ = 5–10.1), the effective number of alleles per locus (*N*_e_ = 3.5–6.0), and the rarefied allelic richness (*A*_r_ = 4.53–6.54), showing the lowest value for Cayo Macho and the highest in Monte Cabaniguán. Observed and expected heterozygosity showed high values for all populations (*H*_*O*_ = 0.464–0.688; *H*_*NEI*_ = 0.704 to 0.830) (Table 1).

**Table 1:** Summary of population genetic parameters for eight *Cyclura nubila nubila* populations from Cuba based on seven microsatellite loci.

Sampling locality	N	*N*_a_	*N*_e_	*A*_r_	*H*_o_	*H*_*NEI*_	Avg *F*_*I*_	95% HPDI
Cabo Corrientes (CCo)	8	5	3.5	4.61	0.464	0.756	0	0
Guanahacabibes (Gu)	18	6.5	3.7	4.88	0.611	0.704	0.1319 ^b^	0.039–0.221
Cayo Alto (CAl)	14	5.5	3.9	4.77	0.602	0.752	0.1832 ^b^	0.077–0.304
Monte Cabaniguán (MCa)	36	10.1	6.0	6.54	0.635	0.830	0.2388 ^a^	0.176–0.304
Cayo Obispo (COb)	20	6.4	4.2	5.16	0.657	0.751	0	0
Cayo Verde (CVe)	23	7.7	4.7	5.59	0.571	0.771	0.1385 ^c^	0–0.265
Cayo Blanquizal (CBl)	26	6.7	4.2	4.95	0.456	0.730	0.3843 ^b^	0.297–0.470
Cayo Macho (CMa)	27	6.1	3.6	4.53	0.688	0.714	0.0627 ^b^	0–0.122
All samples	172	20.2	10.3		0.597	0.884		

Number of genotyped individuals (*N*), mean number of observed (*N*_a_) and mean number of effective alleles per locus (*N*_e_), rarefied allelic richness (*A*_r_), observed heterozygosity (*H*_o_), Nei’s unbiased expected heterozygosity (*H*_*NEI*_), and null allele corrected inbreeding coefficient (Avg *F*_*I*_) for the ^a^ ‘fb’, ^b^ ’f’, ^c^ ‘fn’ models, with posterior 95% probability intervals (95% HPDI).

We estimated the effective population size (*Ne*) per population, which exhibited low values, ranging from 1474.3 for Cayo Verde, 404.4 for Cayo Blanquizal to 82.1 and 24.6 for Cayo Alto and Cayo Macho, respectively (Supplementary Table S3). Most of the confidence intervals (CI) upper bounds were infinite, due to the fact that parametric (and jackknife) methods for computing the *Ne*-associated CI tend to be conservative and cannot be accurately calculated for small sample sizes; however, Waples and Do^31^ indicated in such cases that if adequate data are available, the lower bound of the CI generally will be finite, providing plausible limits. Our inbreeding coefficient DIC results indicated that inbreeding is a prominent component of the best fit model for all populations except Cabo Corrientes and Cayo Obispo (Table 1). In addition, the null allele-corrected inbreeding coefficients (Avg *Fi*; Table 1) were all positive (0.063–0.384), and for which the posterior 95% probability intervals (95% HPDI) can be considered significantly above zero for Guanahacabibes, Cayo Alto, Cayo Blanquizal and Cayo Macho. Three populations showed a contemporary bottleneck signature, based on the observed significant heterozygote excess in comparison to allelic richness: Cayo Alto (Z-test *p* = 0.035; Wilcoxon *p* = 0.016), Cayo Obispo (Z-test *p* = 0.096; Wilcoxon *p* = 0.015), and Cayo Blanquizal (Z-test *p* = 0.058; Wilcoxon *p* = 0.008), while no M-ratio deficiencies were found.

### Genetic structure

We assessed the degree of genetic structure and differentiation among populations with different approaches. Population local *F*_*ST*_s, in which higher values mean greater differentiation of a particular population compared with the others, showed moderate to high *F*_*ST*_ values, the highest for Cayo Macho (0.271) and Cayo Alto (0.251) and lowest for Monte Cabaniguán (0.115) and Cayo Verde (0.157) (Supplementary Table S4). Differentiation among populations was significant and concordant measured either with *Fst* or with Nei’s genetic distance (Supplementary Table S5): highest between Guanahacabibes and Cayo Macho (*Fst* = 0.269; Nei = 2.341), Cabo Corrientes and Cayo Blanquizal (*Fst* = 0.215; Nei = 1.629), and the lowest between Cayo Obispo and Cayo Verde (*Fst* = 0.049; Nei = 0.248) and Cayo Blanquizal and Cayo Verde (*Fst* = 0.091; Nei = 0.41).

**Figure 2:**
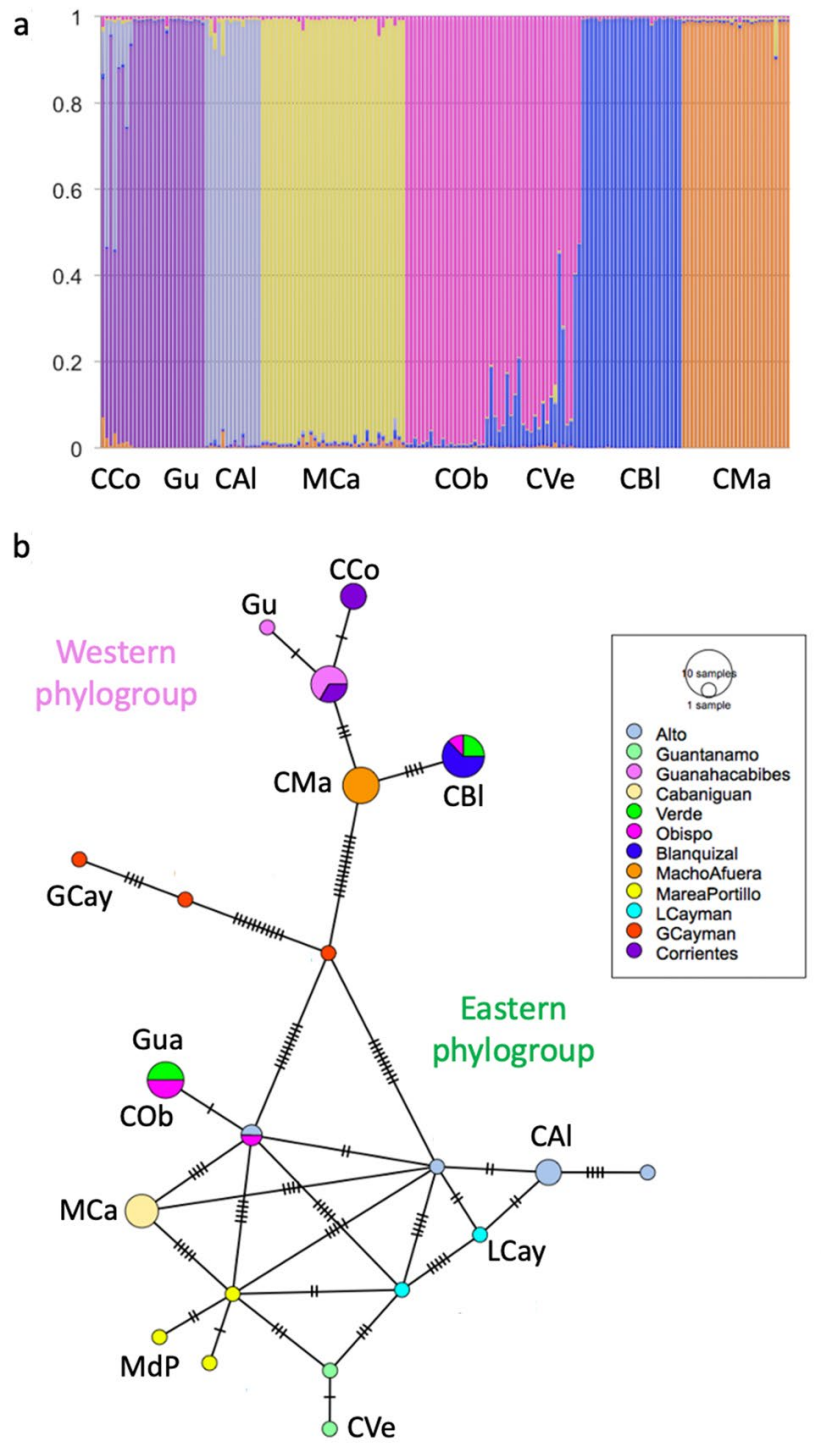
(**a**) Admixture proportions from Structure (*K* = 6) for *Cyclura nubila nubila* from Cuba. Each vertical bar corresponds to an individual and different colors indicate their ancestry to the different clusters. CCo: Cabo Corrientes, Gu: Guanahacabibes, CAl: Cayo Alto, MCa: Monte Cabaniguán, COb: Cayo Obispo, CVe: Cayo Verde, CBl: Cayo Blanquizal, CMa: Cayo Macho. (**b**) Minimum spanning haplotype network for samples of *C. n. nubila* from Cuba, and *C. n. caymanensis* and *C. lewisi*, from Cayman and Little Cayman islands. Circles represent haplotypes and circle size is proportional to haplotype frequency. Color of circles depict the sampling locality or site of origin of samples (see insert). Abbreviations refer to Gu: Guanahacabibes, CCo: Cabo Corrientes, CBl: Cayo Blanquizal, CVe: Cayo Verde, COb: Cayo Obispo, CMa: Cayo Macho, CAl: Cayo Alto, MCa: Monte Cabaniguán, MdP: Marea de Portillo, Gua: Guantánamo, GCay: Grand Cayman, LCay: Little Cayman.

Finally, the genetic structuring and individual admixture ancestry results (Structure) (Fig. 2a) supported six distinct genetic clusters (LnP(K = 6) = − 4788.6), where all individuals were assigned to a cluster with a 91–100% probability of membership. Cabo Corrientes and Guanahacabibes formed a single cluster, Cayo Verde and Cayo Obispo a second one, while the rest of the populations each clustered in a different group. The AMOVA results (Supplementary Table S6) showed that genetic variation resided mainly within individuals (65.6%; *p* > 0.001), while that between genetic clusters and among individuals within populations was 11% and 18.0%, respectively (*p* < 0.001).

As another measure of differentiation and potential inbreeding, we estimated relatedness among individuals. Results showed a high proportion of unrelated individuals in all populations (78.3–100%), followed by half-siblings (7.9–12.8%), siblings (0.6–5.6%) and parent/offspring (0–3.3%). Notably, all 8 individuals from Cabo Corrientes were unrelated (Supplementary Table S7).

### Phylogeography and time of divergence

We successfully amplified an 864 bp ND4/ND5 mitochondrial region for the selected 40 samples (https://github.com/kshaney/Cyclura_Conservation_Units). The model of nucleotide substitution obtained for our dataset was GTR, with gamma shape = 1.015 and proportion of invariant sites I = 0.499. Phylogenetic inference (ML) results showed high bootstrap support values at the species level, while low within *C. nubila nubila*, which is common when evaluating relationships among populations with mostly small branches^32^ (Fig. 1; Supplementary Fig. S1). The topology and divergences were consistent with those reported by^20^.

All of our *C. n. nubila* haplotypes clustered closely with *C. lewisi* from Grand Cayman and *C. nubila caymanensis* from Little Cayman (Fig. 1; Supplementary Fig. S1). Our *C. n. nubila* samples formed two clades with a clear west–east pattern. One clade included mostly western populations (our Western group) and the other (our Eastern group) included the eastern populations on the island. Samples of *C. n. caymanensis* were nested within the Eastern group, whereas those of *C. lewisi* were nested within the Western group. Four samples from GenBank had unconfirmed geographic origins (i.e., listed as “Import NoLocality”, “Maybe” or “Possibly”^33,34^. These samples are more clearly resolved within our tree (Supplementary Fig. S1), thus EU532021 is most likely from Cayo Macho, U66236 from Cayo Alto, and EU532025 and EU532027 are both *C. lewisi* (not *C. n. nubila*).

The topology obtained with Beast for the estimation of the times of divergence was concordant with the ML phylogenetic relationships. In accordance with our estimations, the divergence between the most basal *Cyclura*, *C. pinguis* (Node A in Fig. 1b) and the rest of the species occurred 14.6 million years ago (My) (95% HPD: 12.7–16.5), followed by sequential diversification times at 12.5 My (95% HPD: 9.8–15.03) between the *C. cornuta*, *C. ricordi* and *C. carinata* haplogroups (Node B) and the rest, and at 10.4 My (7.4–13.3) and 8.2 My (5.3–11.1) for the *C. collei* (C) and *C. rileyi* (D) divergences. The time to the most recent common ancestor (TMRCA) for the split between the Eastern and the Western *C. nubila* phylogroups (Node E) was dated at 6.8 My (4.2–9.7), where the former separated from *C. cychlura* (Node F) 5.6 My (3.1–8.4). Within the Eastern phylogroup, the Guantánamo and Marea del Portillo populations (and a *C. n. caymanensis* haplotype; Node G) showed a separation since 3.6 My (1.6–6.8), while within the Western group, the *C. lewisi* haplogroup (Node H) diverged from the Cuban populations 5.7 My (3.4–8.4). Finally, the westernmost populations, Guanahacabibes and Cabo Corrientes (Node I), exhibited a 2.8 My (1.3–4.7) separation from Cayo Macho (Fig. 1b).

Results from the minimum spanning haplotype network showed a pattern of general agreement with the phylogeographic topology (Fig. 2b): the networks for the Western and Eastern *C. nubila* groups were deeply separated (by 34 mutational steps), joined by the Grand Cayman haplotypes (*C. lewisi*). The Western group had few haplotypes, with one shared among Cayo Verde, Blanquizal and Obispo. The Eastern network showed many unique haplotypes belonging to different populations (including those on Little Cayman), although they were interconnected by several mutational steps. Guantánamo and Marea del Portillo were also separated, depicted as terminal haplotypes.

### Conservation units

**Figure 3:**
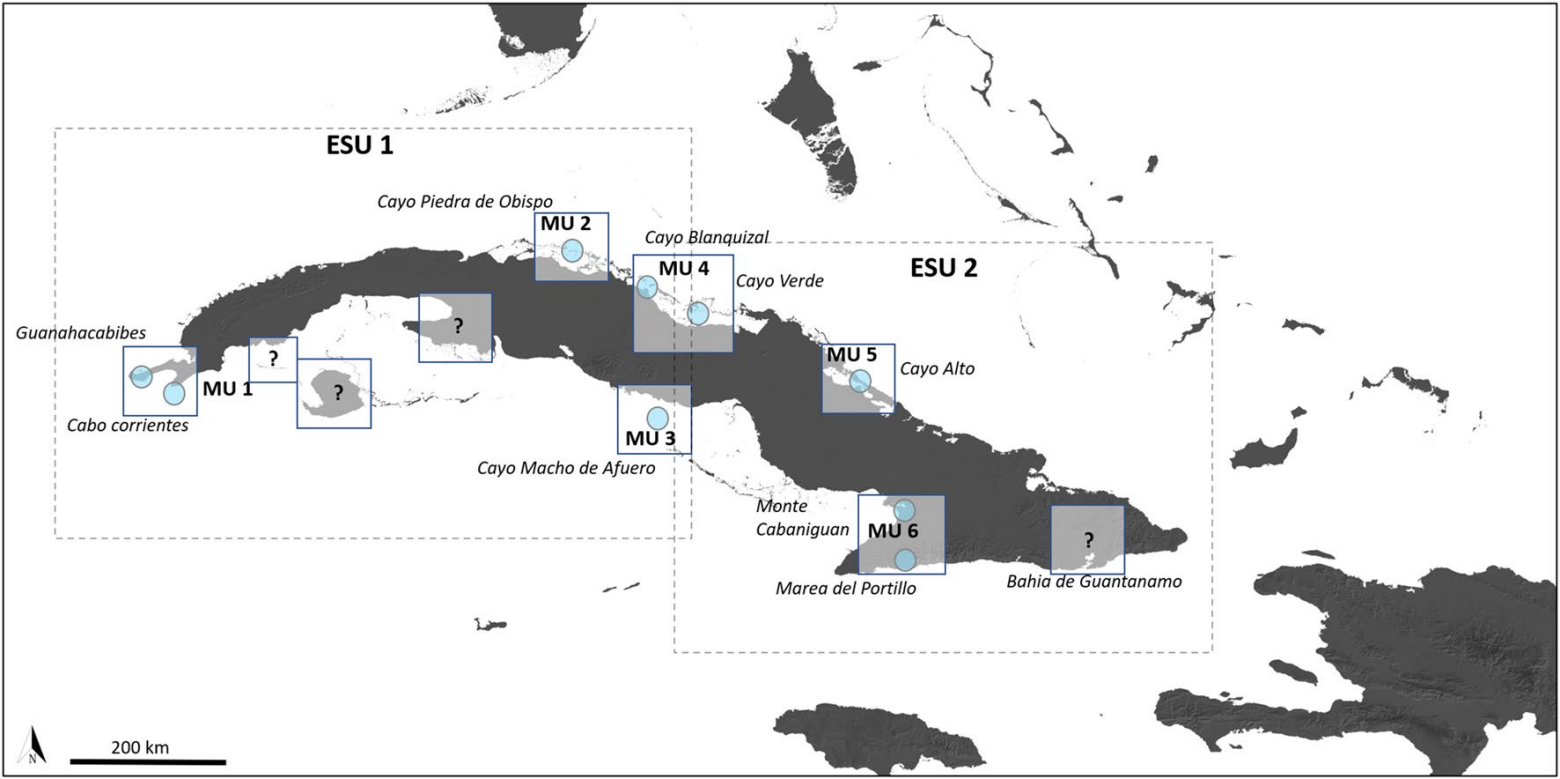
Map delineating Management Units (MUs) and Evolutionary Significant Units (ESUs). There are at least six distinct MUs on Cuba that correspond to our population genetic structure results. MUs marked with “?” represent known populations from other ecological studies which may or may not be genetically distinct MUs, but require genetic confirmation. The two ESUs correspond to the Western and Eastern groups based on mitochondrial DNA (Fig. 1). Map was drawn with QGIS v.3.10 (https://qgis.org/en/site/).

Based on the population structuring obtained from microsatellite loci, we identified at least six different Cuban rock iguana Management Units (MUs) (Fig. 3), while based on the mitochondrial phylogeographic results and the estimated times of divergence, we propose that the Eastern and the Western phylogroups correspond to different Evolutionary Significant Units (ESUs).

## Discussion

### Cuban Rock Iguana MUs and population viability

Rock Iguanas are threatened with extinction mainly because they are harvested for meat and as pets, hence their management should be a high priority. We here present a comprehensive genetic and phylogeographic study for the endangered Cuban Rock Iguana, *Cyclura nubila nubila* within the framework of the West Indian Rock Iguanas (genus *Cyclura*) across the Greater Antilles. Our nuclear genetic data (microsatellite loci) showed significant structure in Cuba across six distinct genetic clusters, exhibiting likely introgression between some populations (Cayo Alto and Cabo corrientes; Cayo Verde and Cayo Blanquizal). This is a classic pattern where the species historically had a wide geographic distribution, which became more isolated contemporarily from a variety of potential factors, like habitat fragmentation, population decrease, or other isolating factors. Although genetic diversity measures are currently moderate to high, it is important to consider that recent bottleneck effects may cause a drop in alleles before a drop in levels of heterozygosity^35^. This means that some of these isolated populations may be suffering the initial stages of a bottleneck that are not yet fully manifested in genetic signatures in this long-lived species.

Based on these considerations, we determined that there should at least be six Cuban Rock Iguana MUs (Fig. 3). Each MU is crucial for the long-term viability of the species and for the ecosystem function they serve at each site. Because they are markedly structured (differentiated) and isolated, composed of predominantly unrelated individuals, most with low effective population sizes, they are prone to genetic drift and inbreeding. Although the estimate of effective population size (*Ne*) is sensitive to sample size and could be under-estimated^30^, it gives a good proxy of the low population numbers for this species^36^. In addition, *Ne* is directly related to the rate of loss of genetic diversity, the potential for bottlenecks, and increased chance of inbreeding^35,37,38^, as already exhibited by some populations of *C. n. nubila*.

The proposed MUs for Cuban Rock Iguanas could persist if migration rates remain stable; nevertheless, our strong evidence of significant differentiation suggests that current migration rates are low. Dispersal may even be further limited by continuing anthropogenic pressures and growing habitat fragmentation. Several of these MUs are currently at risk of extirpation, especially those on Cayo Macho, Cayo Alto, Cabo Corrientes and Cayo Obispo (the first two showed the smallest *Ne*, ranging from only 23–36). Population decline might be exacerbated by the nature of these archipelago systems, where limited habitat, small population size, and unique dispersal and colonization processes, plus potential sea level rise, increase island species vulnerability and susceptibility to extinction^38,39^. Furthermore, endemic insular species generally show proportionately lower genetic variation than island non-endemics^40^. We acknowledge that our sampling is limited and that we did not include other populations that should likely be considered for MU status as well. Undoubtedly, further sampling would aid in confirming the conservation units we suggest. Nonetheless, it is important to highlight that the distribution area of *C. n. nubila* has been reduced in the last 50 years to *ca*. 2000 km^2^, with many localities where it has completely disappeared^23^. Moreover, even back on 1999 Rodríguez-Schettino^24^ had already identified localities (e.g., Pinar del Río, Isla de la Juventud) where the species was extremely hard to find. Thus, our findings are timely and can set the basis for future strategic sampling.

### Considering ESUs

Our phylogeographic results showed two divergent *C. n. nubila* clades*,* delimiting eastern and western Cuban groups. From a widescale biogeographic and phylogeographic perspective, our findings support previous hypotheses that more basal *Cyclura* clades occur in the eastern Caribbean, while more recent divergence occurs along an East to West transect^20^. *Cyclura* populations in Cuba and neighboring islands diverged more recently, as shown by the times of divergence we identified. These patterns are associated with the geological history of Cuba. In the middle Miocene, the western region of Cuba came into contact with central/eastern Cuba when the Havana-Matanzas Channel disappeared^41–43^. These events are in accord with the time of divergence of the Eastern and Western phylogroups we identified (6.8 My ± 4.2–9.7), and with the subsequent events, where the easternmost (Guantánamo, Marea del Portillo) and the westernmost (Guanahacabibes, Cabo Corrientes) populations diverged more recently (Fig. 1b). Other species exhibit this east–west divergence: for instance, the Cuban toad *Peltophryne longinasus*^44^ and the *Eleutherodactylus auriculatus* frog species group^43^. Later, during the Pleistocene, the alternate exposure and submergence of land with glacial cycles, with alternating xeric and mesic environments, resulted in repeated conditions for faunal isolation, speciation, and extinction.

Robertson et al.^45^ emphasized that Evolutionary Significant Units (ESUs) in island systems should be defined by neutral and adaptive loci. Funk et al.^7^ also suggested using robust genomic adaptive and neutral information for defining ESUs. However, the case of threatened rock iguanas from the remainder of the Caribbean archipelago presents an interesting example that may not fit their recommendations. First, given the scarcity of genomic data for this group, it is not currently an option. Second, even if we lack data from adaptive loci to test additional questions, we know that each *Cyclura* population may play a key functional role in seed dispersal and ecosystem functioning. Disappearance of any population would mean the loss of that role, regardless of what adaptive loci tell us about adaptive distinctiveness. Is this enough to qualify geographically isolated populations as ESUs?

Since time is of the essence, and we lack robust adaptive genetic data for most species of *Cyclura*, we suggest it is better to move forward with hypothesized ESUs. Based on our mitochondrial phylogeographic results, we identified distinct Eastern and Western *C. n. nubila* phylogroups (Figs. 1, 3). Indeed, the more historical signal from these data, combined with the estimated times of divergence that indicate a long historical separation of the phylogroups, enable us to propose considering *C. n. nubila* populations as two distinct ESUs (with six MUs) across Cuba. In addition, our broader phylogeographic findings for all *Cyclura* species strengthen the conclusion that each of the taxa of Caribbean rock iguanas, based on their evolutionary and geographic uniqueness, warrant ESU distinction (Supplementary Information Fig. S2).

In addition, although our study was not intended to evaluate the taxonomy of *Cyclura*, we note the paraphyly of *C. nubila* relative to *C. n. caymanensis* and *C. lewisi*. The latter was considered a separate species by Burton^46^ and Malone et al.^20^, based on morphological and molecular data, respectively. However, ITWG^16^) emphasized that additional study is sorely needed. Hence, we believe our findings and proposed ESUs provide valuable additional evolutionary information for the unresolved taxonomy of these iguanas.

### Conservation implications

The Caribbean islands are collectively considered a biodiversity hotspot and have recently been included in the WCS top 14 global priorities strategy^29^. Cuba is especially interesting due to its large size, habitat diversity, and unique array of species, many endemic. However, significant areas of the island’s forests and other natural ecosystems have been converted for agriculture, mining, and ranching^47^. Although all rock iguana MUs identified here overlap with protected areas, many of these areas appear to be quite small, which may limit them from successfully protecting the iguanas. In addition, fragmentation driven by land use changes has further divided natural habitats across the island. Therefore, based on the iguana’s habitat loss, and the genetic differentiation, small population sizes and inbreeding we documented, and considering IUCN guidelines (https://www.iucnredlist.org/assessment/process), we recommend that the IUCN status of *C. n. nubila* is reexamined.

Being able to infer and/or verify the geographic origin of wildlife products (e.g., from traded species) is crucial in conservation^48–50^. Recent methods have been developed that combine genetic, sampling, and statistical methods with that purpose; for instance, Wasser et al.^48^ developed a method that can estimate geographic-specific allele frequencies using microsatellite loci data, successfully identifying the origin of elephant tusks. Another wildlife forensic example used mitochondrial (haplotypes) DNA to identify geographic areas of origin across Southeast Asia of confiscated pangolin scales^49^. Our data could aid in testing the origin of traded Cuban Rock Iguana specimens for which our microsatellite data should allow mapping at a fine resolution. In addition, coarse-scale mapping with the mitochondrial data could at least narrow down identification to the species or subspecies level, as we demonstrated by identifying the locality of the GenBank sequences that had an unknown origin^34^.

## Conclusions

Caribbean ecosystems are rich in biodiversity and endemism, making the management of Cuban Rock iguanas a high priority. Rock Iguanas are an excellent example of an atypical plant-dispersing group whose loss from parts of the Caribbean could have major cascading effects^51^. Beovides-Casas and Mancina^52^ found eight species of plants in *C. n. nubila* fecal samples, the two most common were fruits of *Chrysobalanum icaco* and *Batis maritima*. Hence, as iguana populations decline in Cuba, the loss of their ecological function is also of major concern. We identified at least six *Cyclura nubila nubila* MUs across Cuba, and potentially more could exist, which are nearly completely demographically isolated and with declining population trends. We also defined two ESUs in Cuba, which represent groups with potentially high levels of adaptive genetic variation. Importantly, these conservation units hold the genetic and evolutionary diversity needed to face the challenges of a shifting environment. Climate change is one major threat for species in the Caribbean islands system^44^. The likelihood that a species will persist amid climatic oscillations or sea level rise or disease is higher if several populations remain as intact and connected as possible^9,44^. Our approach shows the importance of considering the historical phylogeographic diversification and contemporary intraspecific genetic variation of species, in addition to their ecological function, for CU management planning.

## Methods

### Sample collection and DNA extraction

Fieldwork was performed at eight sampling localities (populations) across Cuba, two located at protected areas (Biosphere Reserve and Faunal Refuges), two from the mainland inhabiting steep rocks, and the rest from northern offshore cays (Fig. 1a). The institutional review boards of the Universidad de la Habana and Empresa Nacional para la Proteccion de la Flora y Fauna approved the collection and movement of blood samples for the present study, the latter performed via the academic interchange program between Universidad de la Habana-UNAM (‘Red de Macrouniversidades de América Latina y el Caribe’). Field work was performed in compliance with the Guidelines for use of live amphibians and reptiles in field and laboratory research^53^. Sampling was performed during 2004–2006, under the supervision of the Cuban flora and fauna protection authorities at all times; individuals were captured using a lasso (rope) or by excavating them from their shelters, and all animals were released at the site of capture. We obtained a total of 172 individuals (Supplementary Table S1). Sex was determined by cloacal examination for hemipenes and by the degree of development of secondary sexual characters, while individuals that lacked external secondary sexual characters were classified as juveniles. We collected blood samples by a non-invasive method via caudal venipuncture from every individual captured. Blood was stored in buffer (100 mM Tris, 100 mM Na2EDTA, 10 mM NaCl, 1% SDS) at a ratio of 1:2 blood to buffer^27^. DNA was extracted using the AquaPure Genomic DNA kit (Biorad) following the manufacturer’s protocol.

### Microsatellite loci and mtDNA amplification

Microsatellite loci were amplified for all 172 individuals, testing 10 primers developed for *C. n. nubila*^28^: HDZ35, HDZ66, HDZ148, HDZ151, HDZ152, HDZ154, HDZ181, HDZ373, HDZ419, HDZ494. Unfortunately, no samples from Grand Cayman or the Sister Isles were available for this analysis. PCR amplification was carried out in a 10 μl reaction with a final concentration of approximately 50 ng of genomic DNA, 0.2 μM each dNTP, 1.5–2.0 mM MgCl_2_, 10 × reaction PCR buffer (200 mM Tris HCl pH 8.4, 500 mM KCl), 0.5 units of Taq DNA polymerase, and 0.5 pmol/μl of unlabeled reverse primer and fluorescently labeled forward primer. PCR amplification (PTC-100 Thermal Cycler; M.J. Research) was done following the conditions described by^28^. Positive and negative controls were used throughout to ensure correct scoring. PCR products were sequenced at the Roy J. Carver Biotechnology Center-University of Illinois, and the individual genotypes were scored with GeneMapper 3.7^54^.

We amplified mitochondrial DNA with the primers ND4 and LEU (from^55^), which amplify the ND4 and ND5 regions, for a subset of five individuals per sampling locality. PCR reaction was set for 50 μl volume containing: 2 mM MgCl2, 0.2 μM dNTPs, 0.6 μM of each primer, 0.8 µg/µl of BSA and 0.5 units of Taq DNA polymerase. Amplification was done in a PTC-100 Thermal Cycler (M.J. Research), with the following conditions: initial 5 min denaturation at 96 °C, 35 cycles consisting of 96 °C denaturing for 30 s, annealing for 30 s at 49 °C, a 4 min ramp to 72 °C, with a final extension at 72 °C for 1 min^20^. Sanger sequencing was performed by Macrogen-Korea. Sequences obtained were aligned with Geneious 9.0.5 (http://www.geneious.com;^56^) with the Muscle algorithm.

### Genetic diversity and demographic analyses

Based on the microsatellite loci, we examined possible departures from Hardy–Weinberg equilibrium (HWE) with an exact test, and linkage disequilibrium (LD) by a log-likelihood ratio statistic (G-test) using GenePop v.4.0^57^ for each population (sampling locality). We conducted significance tests in GenePop using Fisher’s method, 10,000 dememorizations, 1000 batches, and 10,000 iterations per batch; where necessary, α value was adjusted for multiple comparisons applying Bonferroni correction^58^. The presence of null alleles and stuttering was estimated with the program Micro-Checker v.2.2.3^59^, using a 95% confidence interval and 1000 repetitions. We estimated genetic variability indices, including the observed (*n*_*o*_) and effective (*n*_*e*_) number of alleles, observed heterozygosity (*H*_*o*_) and expected (*H*_*e*_), and Nei’s unbiased expected heterozygosity (*H*_*NEI*_;^60^), with GENALEX v.6^61^ and adegenet in R^62^.

In order to explore demographic patterns, we estimated the effective population size (*Ne*) for populations with a sample size > 10, with NeEstimator v.2.1^63^, based on linkage disequilibrium (LD), a random mating system, and Pcrit (rare-allele critical value) of 0.02. We also calculated inbreeding coefficients in INEst v.2.1^64^, which allows correcting for null alleles. We ran the individual inbreeding model (IMM), testing the ‘null’ model and all combinations of the parameters ‘n’ (null alleles), ‘f’ (inbreeding), and ‘b’ (genotyping failure), using 200,000 Monte Carlo Markov chain (MCMC) iterations and 20,000 burnin; to assess the best model fit for the data, we estimated the Deviance Information Criteria (DIC) for each run. Finally, we evaluated evidence of recent genetic population bottleneck events with INEst, based on two tests: the heterozygosity excess in relation to allelic richness^65^, and the mean ratio of allelic richness to allelic size range deficiencies (M-ratio;^66^). We used the two-phase mutation model and default settings; significance was tested with both a Z-test based on the combined Z scores and a Wilcoxon signed-rank test, with 10,000 permutations.

### Genetic structure, population size and relatedness

We used different approaches to assess the degree of genetic structure and differentiation among populations (sampling localities). First, we used a hierarchical Bayesian method (GESTE v.2.0^67^; to estimate population specific *F*_*ST*_s, where values can be interpreted as a measure of genetic interchange between each local population and the migrant pool (with the rest of the metapopulation). Higher local *F*_*ST*_ values mean greater differentiation of this particular population compared with the others. We used 100,000 iterations with a burn-in of 10,000 and a thinning interval of 100; Cabo Corrientes was not tested due to low sample size. Three independent runs with identical setting values were performed to check for consistency of the estimates. Next, we estimated pairwise differentiation based on *F*_*ST*_ estimated with FSTAT v.2.9.3^68^), and Nei’s genetic distance (*D*_*N*_;^69^) with GenAlex^61^.

In order to estimate the genetic structuring and individual admixture ancestry, we used a Bayesian clustering method (Structure v.2.3,^70^), with values of *K* = 1–9, under an admixture ancestral model and the correlated allele frequencies model. We performed 20 runs for each value of *K*, 100,000 Markov chain Monte Carlo generations, after a burn-in of 50,000 iterations. We used the Evanno’s Δ*K* test to estimate the maximum number of clusters^71^; data were processed with Structure Harvester^72^. In addition, we examined the distribution of the genetic variance using a molecular analysis of variance (AMOVA), considering different hierarchical levels (sampling localities, genetic clusters identified with Structure and individuals), based on *F*_*ST*_ with Arlequin v.3.01^73^; significance was calculated using a non-parametric test with 30,000 permutations of genotypes among populations.

We evaluated relatedness among individuals, another indicator of differentiation at the individual level with ML-Relate^74^, which is designed for microsatellites, is based on maximum likelihood tests, and considers null alleles.

### Mitochondrial sequences and phylogeographic analyses

In order to be able to define conservation units, we performed phylogenetic, phylogeographic and times of divergence analyses based on mitochondrial sequences, both from our *C. n. nubila* individuals and from data for all Caribbean *Cyclura* species. We amplified an 864 bp fragment of the ND4/ND5 mitochondrial region from 40 *C. n. nubila* individuals. To place mitochondrial data into a broader biogeographic context, which is more appropriate for defining CUs (see below), we selected sequences from GenBank (see Supplementary Fig. S1 for the accession numbers) that encompassed the distribution of all *Cyclura* species across the Caribbean^20^. Thus, the final dataset included sequences for *C. n. nubila* from Cuba (our eight sampling sites plus two, from Marea del Portillo and Guantánamo), and from Little Cayman (*C. n. caymanensis*) and Grand Cayman (*C. lewisi*), as well as sequences from *C. pinguis*, *C. cornuta*, *C. ricordi*, *C. carinata*, *C. collei*, *C. rileyi*, and *C. cychlura*. We selected sequences from *Iguana iguana, I. delicatissima* and *Dipsosaurus dorsalis* as outgroups. We estimated the best fitting model of sequence evolution for our dataset, which was subsequently used for maximum-likelihood (ML) phylogenetic analysis, with PhyML 3.0^75^, using a Nearest-Neighbor Interchange and Subtree-Prune and Regraft moves (NNI + SPR) for branch length and topology optimization. We assessed clade support with 1000 non-parametric bootstrap replicates. In order to evaluate relationships among mitochondrial haplotypes, we constructed an unrooted network among unique haplotypes, using PopArt^76^, based on the minimum-spanning method^77^.

### Divergence time estimation

In order to establish the historical time frame of the separation of *Cyclura* species across the Caribbean and for *C. n. nubila* within Cuba, we estimated divergence times (time to the most recent common ancestor, TMRCA) with Beast 1.10.4^78^. Notably, although several studies regarding the systematics of iguanian lizards exist (see^20,79,80^), there are still taxonomic uncertainties, specially at the subspecies level (see ITWG^16^ and references therein), while divergence times for many genera are rather scarce or ambiguous. We were able to obtain two calibration points, the TMRCA for *Dipsosaurus dorsalis* at 20 million years (Ma; 95% = 16–24) and for *Cyclura* spp (14.5 Ma; 95% = 12–16), based on^81–83^. The analysis was done with the complete data set and the outgroup species as used for ML. Parameters included a Yule process tree prior, the GTR + I + G model of evolution across all gene and codon positions, an uncorrelated relaxed-clock dating, 500,000,000 generations sampled every 5000th and 20% of initial generations discarded as burnin. Convergence and stationarity were visualized with Tracer v.1.6 (http://tree.bio.ed.ac.uk/software/tracer/).

### Defining Conservation Units (CUs)

Funk et al.^7^ discussed grouping CUs using genomic datasets; however, the same principles apply to our microsatellite dataset and we follow their suggestions. They explain that ESUs (the largest intraspecific CU) should be defined using a combination of loci under selection and neutral loci because the goal is to delineate populations that have been shaped by neutral and adaptive processes. Conversely, Management Units (MUs), smaller intraspecific CUs, should be assessed with only neutral markers. Therefore, we base MU segment assignment on microsatellite data and ESU segment assignment on mitochondrial data. Additionally, they suggest Bayesian clustering programs like Structure as one potential option for evaluating population subdivision, which we used for evaluating MUs with microsatellite data.

## Ethics declarations

Fieldwork was performed in compliance with the Guidelines for use of live amphibians and reptiles in field and laboratory research^53^.

## Supplementary information

Supplementary Information.

Supplementary Information 2.

## Data Availability

ND4/ND5 *Cyclura nubila nubila* sequences generated in this study are deposited in GitHub (https://github.com/kshaney/Cyclura_Conservation_Units). The microsatellite genotypes are in Supplementary material (Supplementary Table S2).
