# Supplementary material for: Defining intraspecific conservation units in the endemic Cuban Rock Iguanas (Cyclura nubila nubila)
Source: Sci Rep. 2020 Dec 10;10:21607. doi: 10.1038/s41598-020-78664-w (PMC7729961; doi:10.1038/s41598-020-78664-w)
Supplement: Supplementary file 1 — Supplementary Information. [file 41598_2020_78664_MOESM1_ESM.pdf]

**Defining intraspecific conservation units in the endemic Cuban Rock Iguanas (*Cyclura nubila nubila*)**

Kyle J. Shaney, L. Grisell Diaz-Ramirez, Sayra Espindola, Susette Castañeda-Rico, Vicente Berovides-Álvarez, Ella Vázquez-Domínguez

**Supplementary information**
Tables S1, S3-S7
Figures S1-S2

**Table S1.** Number of individuals of *Cyclura nubila nubila* per population (sampling locality) from Cuba used in the study and amplified for microsatellite loci.

| Sampling locality | Samples size | | | | |
|---|---|---|---|---|---|
| | Total | Juveniles | Adults | Females | Males |
| Cabo Corrientes (CCo) | 8 | - | - | - | - |
| Guanahacabibes (Gu) | 18 | 2 | 16 | 1 | 15 |
| Cayo Alto (CAl) | 14 | 8 | 6 | 3 | 3 |
| Monte Cabaniguán (MCa) | 36 | 4 | 16 | 6 | 10 |
| Cayo Obispo (COb) | 20 | 0 | 20 | 10 | 10 |
| Cayo Verde (CVe) | 23 | 4 | 19 | 10 | 9 |
| Cayo Blanquizal (CBl) | 26 | 4 | 22 | 12 | 10 |
| Cayo Macho (CMa) | 27 | 0 | 27 | 13 | 14 |
| All populations sampled | 172 | 22 | 126 | 55 | 71 |

**Table S2.** Genotypes obtained in the present study for *Cyclura nubila nubila* based on seven microsatellite loci [28]. This table is submitted in Supplementary information as an excell (xlsx) document.

**Table S3.** Effective population size (*Ne*) estimates per *Cyclura nubila nubila* population (sampling locality), and globally for Cuba, estimated with NeEstimator v2.1 (Do et al. 2014).

| Population | *Ne* (95% CI) |
|---|---|
| Cabo Corrientes (CCo) | NA |
| Guanahacabibes (Gu) | 206 (30.2 – ∞) |
| Cayo Alto (CAl) | 82.1 (15.8 – ∞) |
| Monte Cabaniguán (MCa) | ∞ (505.5 – ∞) |
| Cayo Obispo (COb) | 69.5 (24.3 – ∞) |
| Cayo Verde (CVe) | 1474.3 (58.8 – ∞) |
| Cayo Blanquizal (CBl) | 404.4 (45.3 – ∞) |
| Cayo Macho (CMa) | 24.6 (13.4 – 61.8) |

**Table S4**. Local $F_{ST}$ values (and 95% confidence interval) for the populations (sampling localities) of *Cyclura nubila nubila* from Cuba, estimated with GESTE v.2.0 (Foll and Gaggiotti, 2006).

| **Localidad** | **$F_{ST}$s** | **95% C.I.** |
|---|---|---|
| Cabo Corrientes (CCo) | 0.226 | 0.146 - 0.319 |
| Guanahacabibes (Gu) | 0.232 | 0.164 - 0.304 |
| Cayo Alto (CAl) | 0.251 | 0.173 - 0.333 |
| Monte Cabaniguán (MCa) | 0.115 | 0.080 - 0.151 |
| Cayo Obispo (COb) | 0.201 | 0.136 - 0.269 |
| Cayo Verde (CVe) | 0.157 | 0.106 - 0.211 |
| Cayo Blanquizal (CBl) | 0.222 | 0.157 - 0.290 |
| Cayo Macho (CMa) | 0.271 | 0.198 - 0.349 |

**Table S5.** Values of genetic differentiation measured with $F_{ST}$ (below diagonal) and Nei's genetic distance (above diagonal) between *Cyclura nubila nubila* populations (sampling localities) from Cuba.

| **Sampling locality** | CCo | Gu | CAl | MCa | COb | CVe | CBl | CMa |
|---|---|---|---|---|---|---|---|---|
| Cabo Corrientes (CCo) | | 0.477 | 0.901 | 1.194 | 1.475 | 1.418 | 1.629 | 1.498 |
| Guanahacabibes (Gu) | 0.103 | | 1.296 | 1.432 | 1.132 | 1.231 | 1.521 | 2.341 |
| Cayo Alto (CAl) | 0.148 | 0.209 | | 0.692 | 1.376 | 1.599 | 1.525 | 1.161 |
| Monte Cabaniguán (MCa) | 0.142 | 0.181 | 0.106 | | | 0.831 | 0.812 | 0.589 |
| Cayo Obispo (COb) | 0.195 | 0.197 | 0.193 | 0.132 | 0.941 | 0.248 | 0.459 | 1.359 |
| Cayo Verde (CVe) | 0.183 | 0.196 | 0.196 | 0.117 | 0.049 | | 0.415 | 1.055 |
| Cayo Blanquizal (CBl) | 0.215 | 0.233 | 0.211 | 0.130 | 0.111 | 0.091 | | 1.244 |
| Cayo Macho (CMa) | 0.217 | 0.269 | 0.196 | 0.112 | 0.211 | 0.180 | 0.211 | |

**Table S6.** Analysis of Molecular Variance (AMOVA) based on $F_{ST}$ for *Cyclura nubila nubila* from Cuba.

| **Source of variation** | **df** | **Sum of squares** | **Variance components** | **Porcentage of variation** | ***P*** |
|---|---|---|---|---|---|
| Among genetic clusters | 5 | 153.64 | 0.3514 | 11.04 | >0.001 |
| Among populations within genetic clusters | 2 | 17.41 | 0.1683 | 5.29 | >0.001 |
| Among individuals within populations | 164 | 531.12 | 0.5742 | 18.03 | >0.001 |
| Within individuals | 172 | 359.50 | 2.090 | 65.64 | >0.001 |

**Table S7**. Proportion (%) of relatedness of individuals within each population (sampling locality) for *Cyclura nubila nubila* from Cuba. Cabo Corrientes (CCo), Guanahacabibes (Gu), Cayo Alto (CAl), Monte Cabaniguán (MCa), Cayo Obispo (COb), Cayo Verde (CVe), Cayo Blanquizal (CBl), Cayo Macho (CMa)

| **Relatedness** | **CCo** | **Gu** | **CAl** | **MCa** | **COb** | **CVe** | **CBl** | **CMa** |
|---|---|---|---|---|---|---|---|---|
| Parent/offspring | 0 | 3.27 | 0 | 0.32 | 1.05 | 0.4 | 0.31 | 2.85 |
| Siblings | 0 | 0.65 | 1.1 | 1.4 | 3.16 | 2.37 | 2.46 | 5.98 |
| Half-siblings | 0 | 11.11 | 10.99 | 8.88 | 7.89 | 11.07 | 9.54 | 12.82 |
| Unrelated | 100 | 84.97 | 87.91 | 89.37 | 87.89 | 86.17 | 87.69 | 78.35 |

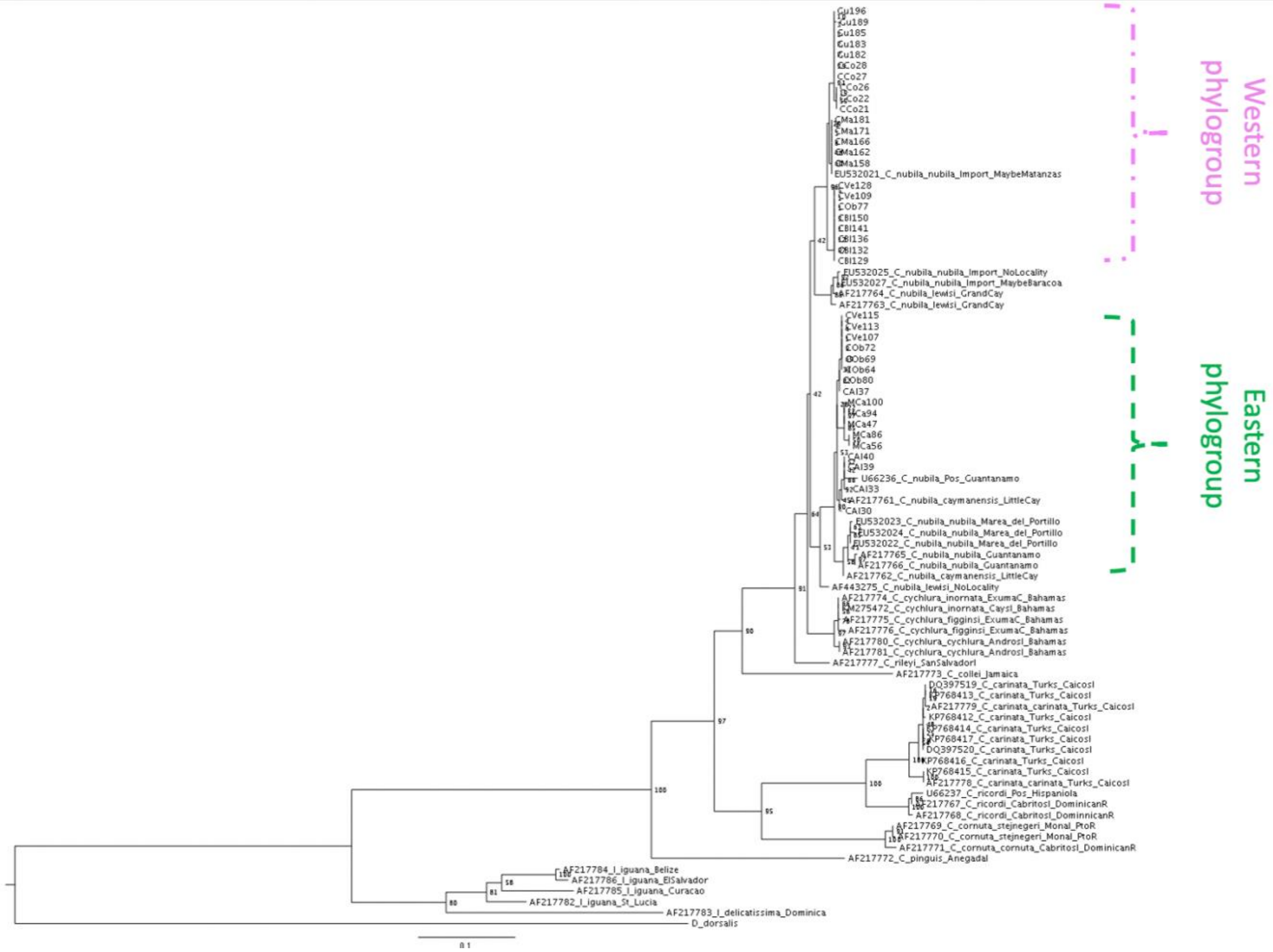


**Figure S1.** Maximum likelihood phylogenetic tree of *Cyclura nubila nubila* from Cuba and *Cyclura* species and subspecies across the Caribbean (*C. n. caymanensis*, *C. lewisi*, *C. pinguis*, *C. cornuta*, *C. ricordi*, *C. carinata*, *C. collei*, *C. rileyi*, *C. cychlura*). *Iguana iguana*, *I. delicatissima* and *Dipsosaurus dorsalis* used as outgroups, based on the ND4/ND5 mitochondrial region. The scale bar represents substitutions per site.

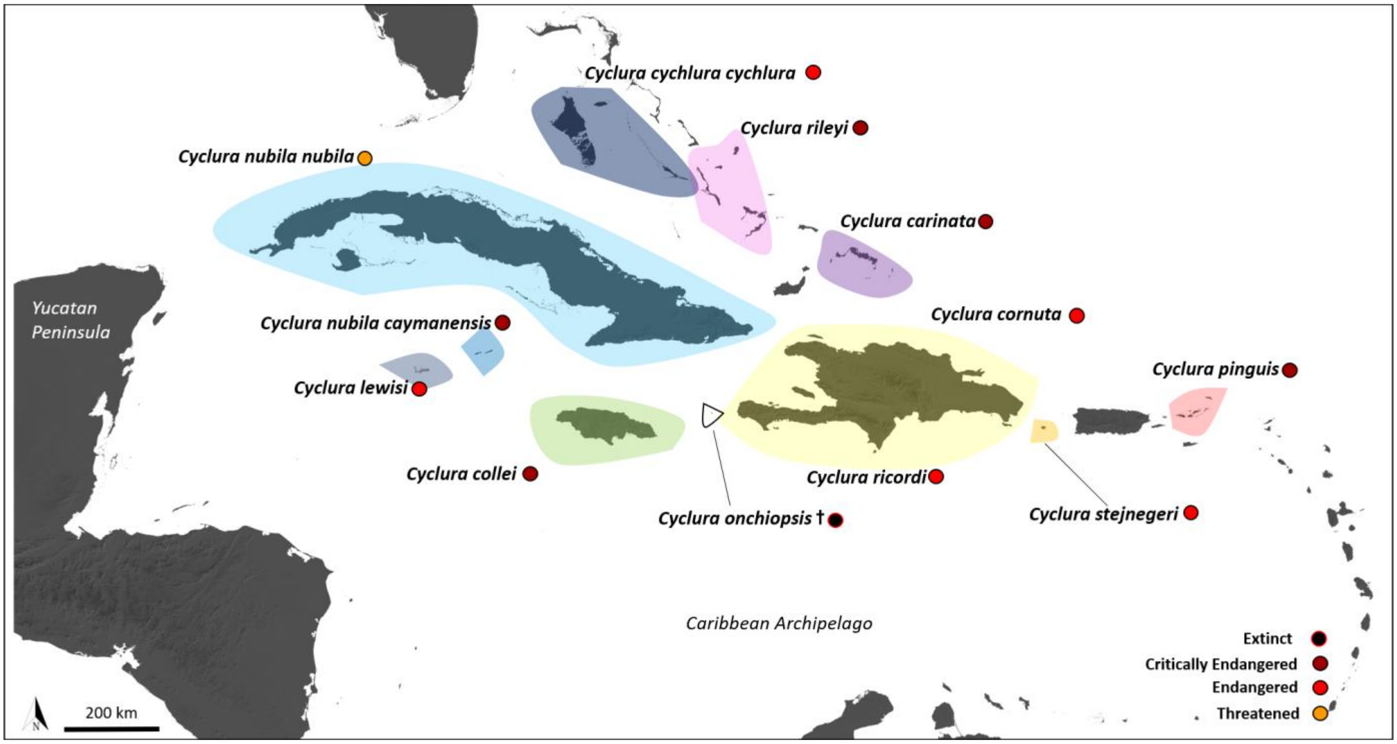


**Figure S2.** Map of the relative distributions of all species within the genus *Cyclura*, where exact distributional limits require additional survey effort. The IUCN Red List status of each species is shown. Our findings support that each species and subspecies merit be considered an Evolutionary Significant Unit (ESU). *Cyclura cornuta* and *C. ricordi* overlap in distribution and both their ranges are represented by the same color encompassing Hispaniola island (yellow). Map was drawn with QGIS v.3.10 (https://qgis.org/en/site/).
